# Immaterial and monetary gifts in economic transactions: evidence from the field

**DOI:** 10.1007/s10683-017-9536-1

**Published:** 2017-08-02

**Authors:** Michael Kirchler, Stefan Palan

**Affiliations:** 1 Department of Banking and Finance, University of Innsbruck, Universitätsstrasse 15, 6020 Innsbruck, Austria; 2 Department of Economics, Centre for Finance, University of Gothenburg, Vasagatan 1, 40530 Gothenburg, Sweden; 3 Department of Banking and Finance, University of Graz, Universitätsstrasse 15, 8010 Graz, Austria

**Keywords:** Gift exchange, Reciprocity, Immaterial gifts, Natural field experiment, D01, D03

## Abstract

Reciprocation of monetary gifts is well-understood in economics. In contrast, there is little research on reciprocal behavior following *immaterial gifts* like compliments. We narrow this gap and investigate how employees reciprocate after receiving immaterial gifts and material gifts over time. We purchase (1) ice cream from fast food restaurants, and (2) durum doner, a common lunch snack, from independent vendors. Prior to the food’s preparation, we either compliment or tip the salesperson. We find that salespersons reciprocate compliments with higher product weight than in a control treatment. Importantly, this reciprocal behavior following immaterial gifts grows over repeated transactions. Tips, in contrast, have a stronger level effect which does not change over time.

## Introduction and literature

It is indisputable that employees appreciate monetary rewards. They expend additional effort when they receive wages exceeding a theoretical minimum wage. Akerlof (1982), for instance, argues that higher wages serve as “gifts” for employees, who reciprocate with higher effort. Laboratory evidence is broadly consonant, showing that higher wages lead to higher effort by employees (Fehr et al. 1993; Fehr and Falk 1999; Fehr and Gächter 2000; Gächter and Falk 2002; Charness 2004). Evidence from field experiments is mainly supportive as well. Most studies report that employees expend more effort following monetary gifts (Falk 2007; Maréchal and Thöni 2007; Kube et al. 2012; Currie et al. 2013; Cohn et al. 2015), while there is some evidence showing that the effects can be temporary (Gneezy and List 2006).

Surprisingly, the role of purely immaterial gifts—e.g., private compliments and individual expressions of appreciation and respect—in economic contexts is less clear. This is remarkable, as the desire for approval and being esteemed is deeply rooted in human behavior, likely because esteem is associated with material and reproductive benefits (Fershtman and Weiss 1998; Fessler 2004). Moreover, reciprocating positive immaterial stimuli may have evolved as an evolutionarily stable strategy, because it signaled potential for future cooperation (Gintis et al. 2003). Theoretical studies postulate that expressions of esteem may increase the recipients utility and that the recipient will then reciprocate with additional effort (Brennan and Pettit 2004; Ellingsen and Johannesson 2007, 2008, 2011). However, evidence from the field is rather scarce. In the behavioral management literature the distinct concept of public social recognition is conjectured to be an important performance reinforcer beside money (Haynes et al. 1982; Bandura 1986; Markham et al. 2002; Stajkovic and Luthans 2003). The behavioral labor market literature also reveals some evidence that public recognition programs and awards positively influence work effort (Kosfeld and Neckermann 2011; Bradler et al. 2014).^1^ However, such programs and awards include an extrinsic component in that they are awarded publicly, and thus provide the recipient with status through publicity (Frey 2007).

Immaterial gifts as defined in our study—i.e., private compliments and individual expressions of appreciation and respect—*do not contain a public component*. The effects of such compliments or expressions of esteem are barely investigated even though this form of gift exchange is fundamental to everyday economic interactions. It occurs constantly between employees and employers, between employees at different hierarchy levels, and between salespersons and customers. To the best of our knowledge, Bradler and Neckermann (2016) is among the closest studies to ours as they investigate the role of singular nonfinancial gifts (Thank You cards) and gifts that combine financial and nonfinancial elements. They find that Thank You cards that signal worker appreciation induce reciprocity and they report interaction effects between money and appreciation when combined with a “personal touch” (i.e., a handmade element like money folded as a bow tie). In another study related to ours, Kube et al. (2012) show that non-monetary gifts have a more pronounced impact on work effort than monetary gifts of equivalent value. However, the non-monetary gifts in both studies are still material (i.e., a thermos bottle and a Thank You card) and thereby different from our concept of immaterial gifts (i.e., private compliments).^2^

Moreover, research on the influence of reciprocal behavior when gifts are given repeatedly over time is rather scarce. Gneezy and List (2006) show that reciprocal effects following *one monetary gift* can be temporary and fade out over time. Ockenfels et al. (2015) extend their analyses and show that work performance is higher for the same total wage when wage is increased in *two* steps as opposed to a single increase. Importantly, the impact of the *repeated provision of immaterial gifts* is unknown.

In addition, material and particularly immaterial gift exchange situations have nearly exclusively been investigated in classical employer–employee relationships, raising the importance of robustness in other domains. We identify customer–salesperson interactions as ideal for analyzing the effects of repeated immaterial and material gift exchange. From a consumer perspective, interactions with salespersons are highly relevant as they occur frequently (i.e., in some cases multiple times a day) and extra effort/kindness from the salesperson is valued greatly.

In this paper we narrow these research gaps by reporting results from two natural field experiments (Harrison and List 2004), both of which involve a salesperson preparing a food item following a customer (experimenter) order. We study whether immaterial gifts in the form of a private compliment (treatment COMPLIMENT) and monetary gifts in the form of a tip (treatment TIP)—both provided prior to the product’s preparation—trigger reciprocal behavior from the salesperson. The third treatment, NORMAL, serves as a benchmark. In the first experiment, we collected one-shot data for purchases of ice cream from McDonald’s restaurants, investigating salespersons’ reciprocation induced by our experimental treatments. We quantify the level of salespersons’ reciprocal behavior by measuring the food items’ weights. We further study the impact of repeated provision of immaterial and of monetary gifts by collecting data for repeated purchases of doner durum from independent vendors. Here, the experimenters visited the same salesperson on five consecutive days, exploring how the observed effects develop over time.^3^

Translating the idea of Ellingsen and Johannesson (2007) on social esteem to our setting, a salesperson’s utility depends both on her income and on her pride from being esteemed by the consumer. We hypothesize that making compliments leads to increased salesperson utility and therefore to increased reciprocal behavior. The salesperson is made to feel proud of what she is doing and exchanges kindness (measured by product weight) for given esteem, yielding our first research question.^4^

RQ1: Does an immaterial gift in the form of a compliment provided by the consumer trigger increased salesperson kindness compared to “normal” consumer–salesperson interactions?

Based on the literature outlined above, we expect a tip in advance to also increase a salesperson’s utility, which she reciprocates with increased product weight. This exchange of greater salesperson kindness for extra money underlies our second research question.

RQ2: Does a monetary gift by the consumer trigger increased salesperson kindness compared to “normal” transactions in consumer–salesperson interactions?

As mentioned, Gneezy and List (2006) and Ockenfels et al. (2015) show the importance of investigating how reciprocity develops over time. While the former find that in a labor market setting reciprocal behavior triggered by one monetary gifts is temporary, the latter report that work performance is higher for the same total wage when wage is increased in two steps rather than once. We address this issue and extend it by providing five immaterial or five monetary gifts in five consecutive transactions over the same number of working days. This design allows us to answer our third research question.

RQ3: Do the effects of repeated immaterial and monetary gifts change over time?

We find that both immaterial gifts (compliments) and material gifts (tips)—given in advance—induce positive reciprocity, i.e., salespersons provide more product weight. While monetary gifts trigger a larger level effect, only the effect of immaterial gifts increases significantly over repeated interactions. Finally, we show that the increase in product weight does not necessarily suffice to compensate the customer for the increased cost of the tip, which means that immaterial gifts are more effective when accounting for transaction costs (i.e., the purchase price and the tip) in our settings.

With our approach we extend the literature along four dimensions. First, we explore reactions to material gifts and particularly to immaterial gifts in the same settings (ice cream and doner), making them comparable. Importantly, our approach of defining immaterial gifts differs from those of Kosfeld and Neckermann (2011), Bradler et al. (2014), Bradler and Neckermann (2016) and the literature on social recognition programs. The “Thank You” cards and award certificates employees receive in these studies have both a material and an (immaterial) award component. A card or a certificate can be preserved and may provide utility at a later time. It can also be displayed to serve as a public signal to others. A private compliment like in our study is entirely immaterial, cannot be “stored”, cannot be used as physical evidence for impressing others, and thereby expresses respect directly (and usually only) to the recipient. Second, we investigate both types of gifts in natural consumer–salesperson interactions in everyday life situations. We consider this an important aspect of our study as we bring monetary and immaterial gift exchange situations to customer–salesperson interactions, testing for robustness of the results from the literature, which largely derive from employer–employee interactions. We believe that consumer–salesperson interactions are of high importance, because they occur very frequently (i.e., in some cases multiple times a day) and monetary and immaterial incentives (i.e., compliments) play a major role. Third, we use a repeated setting to analyze how monetary and immaterial gifts work over time. We outline the importance of repeated gift exchange situations by showing that reciprocal behavior following immaterial gifts gets stronger over time. Fourth, we analyze the robustness of our findings by investigating whether results replicate from the first to the second setting, i.e., from the ice cream to the doner setting.

## Ice cream setting

Both settings of this study (ICECREAM and DONER) were carefully selected to fulfill the following four requirements, ensuring a high degree of experimental control. First, the entire consumer–salesperson interaction, including accepting the order, preparing the product, and accepting the payment is attended to by a single salesperson. Many comparable settings are characterized by a division of labor on the salesperson side. For instance, a waiter in a restaurant has to rely on the barkeeper and on the kitchen staff when aiming to provide high quality drinks and food in minimum time. Second, the amount of additional food provided is measurable. One might get better treatment with tipping or complimenting in advance in many service settings—e.g., getting a massage, or a haircut—, yet this better treatment may not be easily measurable. Third, the salesperson has the discretion to choose a higher than normal amount of food. Many services are standardized with no possibility for the salesperson to intervene by adding any additional benefit. For example, beverages are frequently filled to the brim, with no chance to add any extra. Finally, the consumer–salesperson interaction reflects an everyday life situation. Going to snack bars or restaurants to order ice cream or durum doner is a common occurrence, even when repeated on five consecutive days.

### Experimental procedure—ice cream

**Fig. 1:**
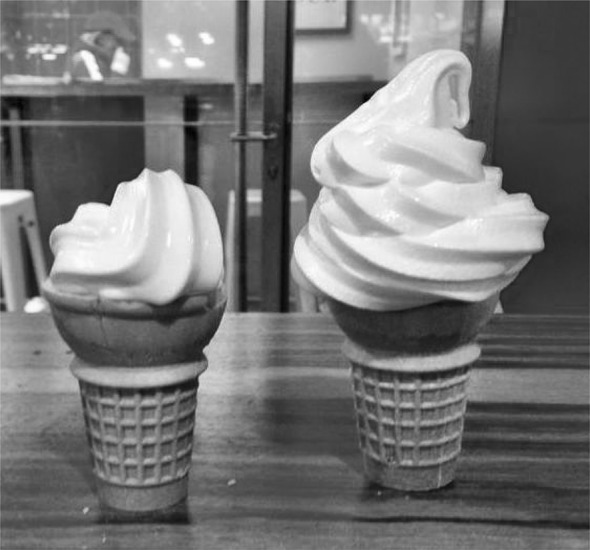
Sample photo of two ice cream cones

In our first experimental setting, ICECREAM, the experimenters ordered ice cream in McDonald’s restaurants. The restaurant clerk takes the client’s order in a face-to-face interaction, accepts payment, fills the ice cream into a cone and hands it over to the customer. Since the salesperson only has to press and release a button to start and end the filling process, it is easy for her to vary the amount of ice cream per cone. See Fig. 1 for a sample photo of two ice cream cones purchased during the experiment.

In treatment NORMAL the experimenter ordered using the standardized wording (translated from German): “One vanilla cone without topping, to take away please.” This standardization ensured that the products were comparable and extra benefit provided by the salesperson could be clearly measured and quantified. The only way in which the salesperson could provide such extra benefit in the interaction (apart from, e.g., being particularly friendly, or gifting the consumer with complimentary goods) was to increase the amount of ice cream. We consciously refrained from ordering toppings, because they might have added noise. The experimenters also did not start conversations with the salespeople. In case they were asked questions, they answered naturally but succinctly.

Treatment COMPLIMENT was identical to NORMAL, but the experimenter made a compliment about the product, prior to the product’s preparation. The standardized wording was: “One vanilla cone without topping, to take away please. You have the best ice cream in town.”

Treatment TIP was identical to treatment NORMAL except that the experimenter gave a tip to the salesperson. As a percentage of price, tips varied between 10.0 and 14.3%, with a mean of 13.1%. This was due to the requirement of total payment amounts being multiples of €0.10 in order to remain inconspicuous. The experimenter took great care to ensure that the tip was recognized by the salesperson at the time the order was placed. The experimenter put the product price plus the tip on the counter and simultaneously augmented the order by adding “The rest is for you” to the standardized wording. The experimenter also chose the coins handed over to the salesperson such that the latter could see that the tipping was intentional and not caused by, e.g., rounding to the nearest integer amount.^5^

All observations were collected by three male experimenters aged 23–25 years. We randomized experimenter roles and arrival orders to control for experimenter fixed effects. Specifically, each experimenter ordered 12 times each using the procedures for treatments NORMAL, COMPLIMENT and TIP. We also randomized the order in which experimenters entered restaurants, such that each experimenter was the first to enter a restaurant 12 times, was second 12 times and was last 12 times. Finally, we randomized the order in which the experimenter playing a specific role entered a restaurant, such that the experimenter in treatment NORMAL was the first to enter a restaurant 12 times, was second 12 times and was last 12 times (and the same for treatments COMPLIMENT and TIP).^6^ Importantly, all three experimenters always interacted with the same salesperson in a given restaurant. Although the experimenters were never present in the restaurant at the same time, they entered in short intervals of 5 min, allowing to control for salesperson and time effects simultaneously. We selected 36 salespeople in 13 different McDonald’s outlets in Innsbruck, Austria, and Munich, Germany, for a total of 108 observations (see the “Appendix” for details on the experimental procedure).

After conclusion of each transaction, the experimenter stepped outside the restaurant to a place where he was not visible from inside and immediately weighed the ice cream cone on small letter scales which he carried in a backpack. He noted the weight and usually gave the ice cream to a passerby. The experimenter then filled in a form recording details about the transaction. These were the restaurant ID, product price, and tip amount (if any), as well as salesperson characteristics like gender, estimated age and ethnicity.

### Results—ice cream

In the top panel of Table 1 we show raw ice cream weight in grams. It is evident that average ice cream cone weights differ markedly between salespeople and treatments. Across treatments raw weight varies from 70 to 167 g, with a mean of 117 g. In particular, ice cream weight is on average highest in treatment TIP and lowest in treatment NORMAL. Results of treatment TIP change when accounting for the money spent (purchase price plus tip) in the bottom panel of Table 1.

**Table 1:** Descriptive statistics: mean, median, standard deviation, minimum, and maximum of raw ice cream cone weights across treatments in grams (top panel) and in grams per euro spent (bottom panel)

Treatment	Observations	Mean	Median	SD	Min	Max
*Raw ice cream weight* (g)						
NORMAL	36	106.03	103.50	18.78	69.60	153.15
COMPLIMENT	36	117.20	113.18	20.55	77.20	163.25
TIP	36	126.62	125.40	19.41	85.20	166.80
*Raw ice cream weight* (g) *per euro spent*						
NORMAL	36	132.26	130.79	26.44	78.20	188.93
COMPLIMENT	36	146.27	146.53	29.49	86.74	233.21
TIP	36	139.96	138.80	27.15	85.20	208.50

**Fig. 2:**
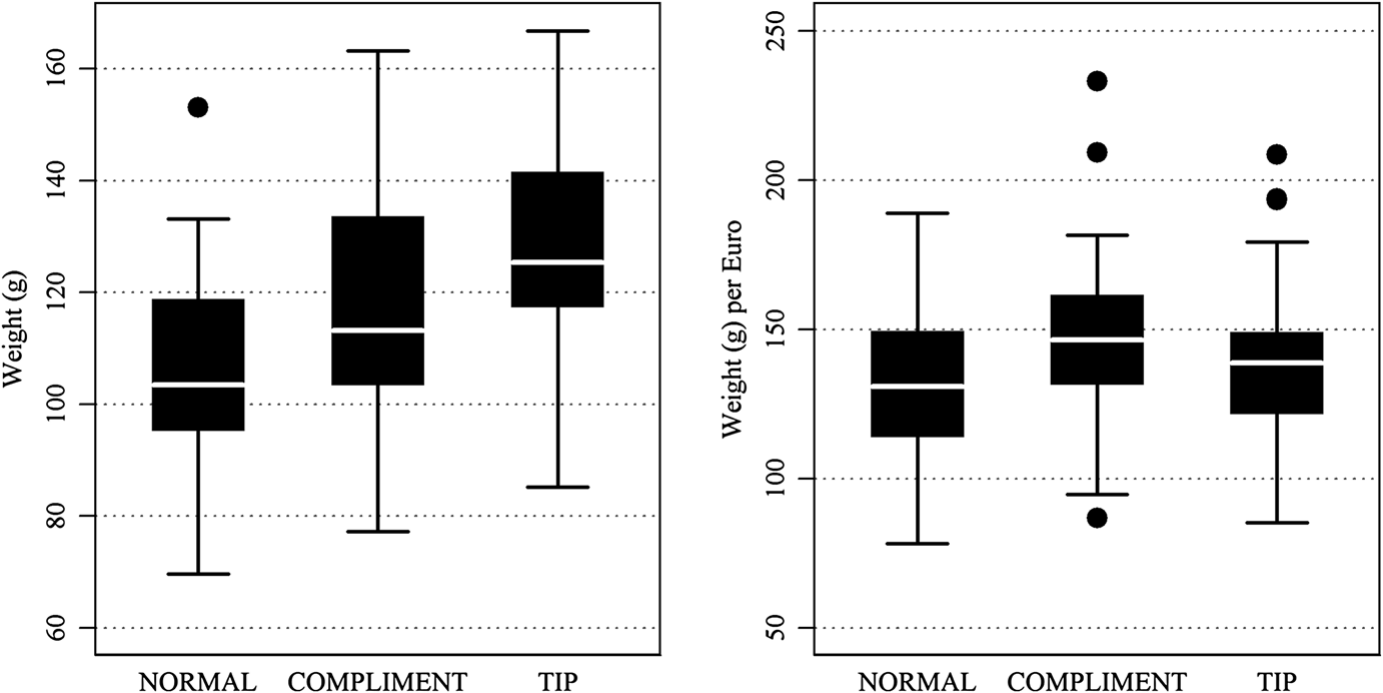
Mean ice cream weight in grams (*left panel*) and in grams per euro (*right panel*) across treatments NORMAL, COMPLIMENT and TIP

In addition, Fig. 2 depicts box plots of ice cream weight in grams and in grams per Euro spent (see Fig. 5 in the “Appendix” for a robustness check using baseline-normalized weight).

On aggregate we find that a compliment increases mean raw cone weight by 11.2 g (two-tailed, pairwise *t* test of raw weights, COMPLIMENT vs. NORMAL: *t*(35) = 4.9802, *p* = 0.0000) and a tip in advance by 20.6 g (TIP vs. NORMAL: *t*(35) = 6.3761, *p* = 0.0000; TIP vs. COMPLIMENT: *t*(35) = 3.0996, *p* = 0.0038). In our regression model in Table 2 we observe both differences to the baseline to be highly significant even while using salesperson fixed effects and experimenter dummy variables (models 1 and 2).^7^^,^ 8 When we analyze ice cream weight per euro spent (models 1M and 2M), we still find a highly significantly positive effect for treatment COMPLIMENT, but only a weakly significant effect for treatment TIP. The net benefit to the customer after accounting for the cost of the tip shrinks to approximately 7.1% or 7.7 g in treatment TIP.

**Table 2:** Panel regressions of ice cream cone weight in grams (models 1, 2) and cone weight in grams per euro spent (models 1M, 2M) across treatments

Regressors	Model 1	Model 2	Model 1M	Model 2M
COMPLIMENT	11.172	11.172	14.008	14.008
	(2.265)***	(2.268)***	(2.920)***	(2.909)***
TIP	20.589	20.589	7.695	7.695
	(3.260)***	(3.286)***	(3.969)*	(4.010)*
Experimenter dummies		Yes		Yes
Constant	106.029	105.438	132.262	131.215
	(1.567)***	(2.305)***	(1.959)***	(2.831)***
*R* ^2^ between	0.42	0.43	0.18	0.19
*R* ^2^ within	0.00	0.01		0.00
*R* ^2^ overall	0.16	0.16	0.04	0.04
*N*	108	108	108	108

Panel regression with salesperson fixed effects. Standard errors clustered at the salesperson level (in parentheses)

* *p* < 0.1; ** *p* < 0.05; *** *p* < 0.01

## Doner setting

We conduct a second experiment to investigate (1) how reciprocity in response to both immaterial and material gifts develops over time, and (2) whether the observed effects are robust to using a setting where providing extra food requires greater effort by the salesperson.^9^

### Experimental procedure—doner

**Fig. 3:**
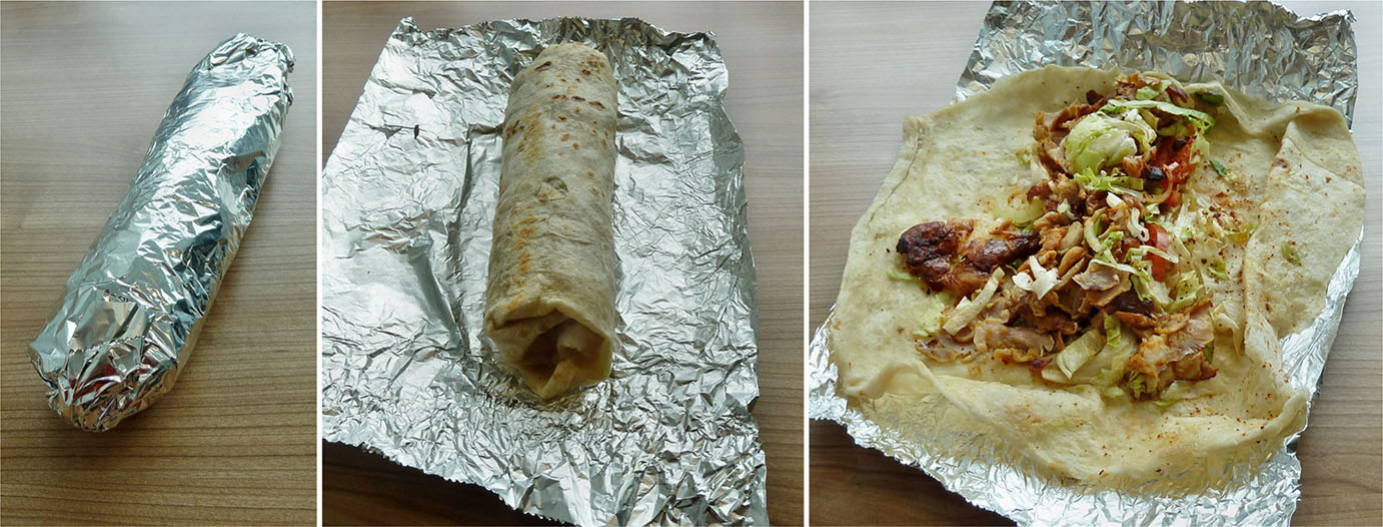
Sample photos of a durum doner: wrapped in foil (*left*), wrapped without foil (*middle*) and unwrapped (*right*)

In setting DONER, the experimenters ordered durum doner in restaurants and snack bars. A durum doner, pictured in Fig. 3, is a dish of Turkish origin, made of meat roasted on a vertical spit, and served in a wrap. Providing extra food in the ice cream setting only requires the salesperson to press the ice cream machine’s button just a few fractions of a second longer. In the doner setting, however, the salesperson has to deliberately transfer additional amounts of food to the wrap using tongs. Furthermore, the wrap’s capacity usually binds more tightly (in percentage terms) than the ice cream cones’. We thus consider this second setting to be a challenging robustness check for the findings from the first.

Experimenters bought durum doner from the same salesperson on five consecutive days. An individual experimenter’s role (i.e., treatments NORMAL, COMPLIMENT, TIP) was fixed for each salesperson and we again randomized the experiment (see below for details). We conducted the experiment in Graz (GRZ) and Innsbruck (IBK), Austria, and in Munich (MUC), Germany. In each of the three cities, three experimenters visited the same 18 restaurants, yielding a total of 54 restaurants and 801 individual observations.^10^

Table 3 shows the number of observations per treatment and visit. In some restaurants, salespersons changed during the observation period, such that we obtained more observations for early than for late visits with a particular salesperson.^11^

**Table 3:** Number of observations for each visit and treatment in experiment DONER

Visit	NORMAL	COMPLIMENT	TIP	Sum
*Number of observations*				
1	69	69	69	207
2	58	57	58	173
3	53	52	54	159
4	48	44	47	139
5	42	40	41	123
Sum	270	262	269	801

All selected restaurants are organized in such a way that the salesperson takes the client’s order personally, collects the money, prepares the doner in front of the client and hands over the finished product. Thus, the entire service process is executed by one and the same salesperson. We employed eight male experimenters of similar age (22–26 years), but only three of them collected data in a particular town. One experimenter was active in two towns. Each experimenter ordered one durum doner from a given salesperson on each of *five consecutive days*. An individual experimenter’s role (i.e., treatments NORMAL, COMPLIMENT or TIP) was fixed for each salesperson. We furthermore strove to ensure that all experimenters always interacted with one and the same salesperson in a given restaurant. We thus designed the experiment to obtain 15 observations per salesperson (three treatments/experimenters, and five observations each). To control for experimenter fixed effects we randomized the experiment. We applied each of the six possible assignments of treatments to experimenters (NORMAL-TIP-COMPLIMENT or NTC, NCT, TNC, TCN, CNT, CTN) three times to cover the 18 restaurants in each town. Thus, each experimenter played each role six times (for five visits each) in each town. Again, the experimenters entered each restaurant independently from each other and were never present in the restaurant at the same time.^12^

After exiting the restaurant, the experimenter weighted the doner as is, i.e., including the tin foil the doner was wrapped in (the weight is negligible in comparison to the product weight and does not vary systematically across treatments). The experimenter noted the weight and put the doner into his backpack for later hand-over to a charitable agency.^13^ The experimenter then recorded the date, time, restaurant, product price, and tip amount (if any), as well as salesperson characteristics as in ICECREAM. The final experimenter to interact with any salesperson also inquired whether the salesperson was the owner or an employee of the restaurant. The experimenters furthermore took pictures of all durum doner during the weighing procedure (data available upon request).

In treatment NORMAL the experimenter used the standardized wording (translated from German): “One durum doner without sauce, to take away please.” The only way in which the salesperson could provide extra benefit in the interaction (apart from, e.g., being particularly friendly, or gifting the consumer with complimentary goods) was to increase the amount of meat or other ingredients, since the durum wraps are standardized. We deliberately refrained from ordering sauce, because it has high relative density and might add noise. One could argue that getting more doner weight may not be considered beneficial by every customer. However, getting something extra is a typical act of kindness in the service industry (e.g., receiving an additional drink, or a free starter in restaurants).

The five standardized wordings in treatment COMPLIMENT, which were used in randomized order, were:

1. “One durum doner without sauce, to take away please. You have the best durum doner in town.”;
2. “[...] It tastes best at your place.”;
3. “[...] By the way, your durum doner tastes great.”;
4. “[...] I never had a better durum doner than at your place.”;
5. “[...] There is no place where the durum doner tastes better.”

Treatment TIP was conducted analogously to the corresponding treatment in ICECREAM. As a percentage of price, tips varied between 8.1 and 10.3%, with a mean of 9.2%.

### Results—doner

**Fig. 4:**
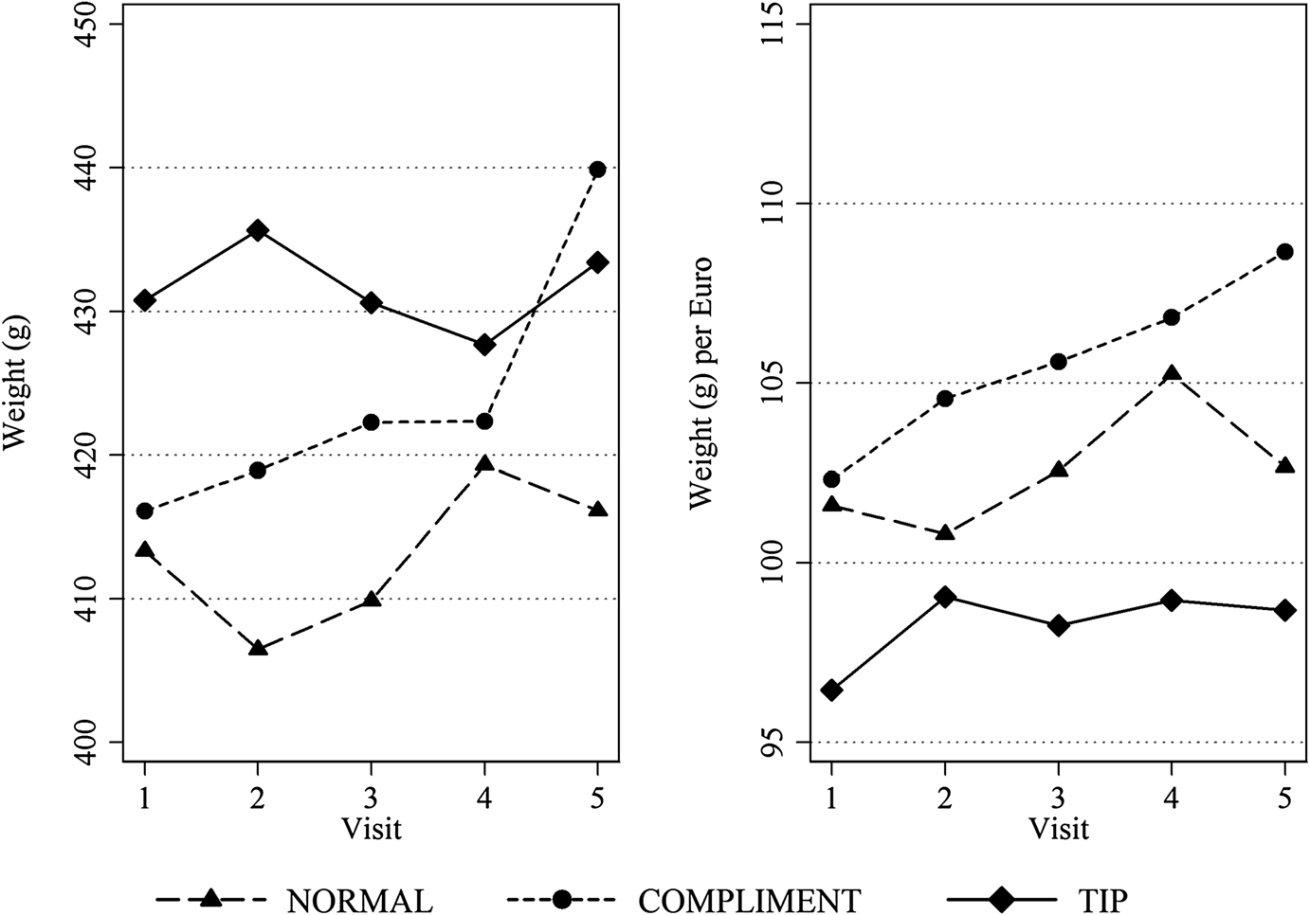
Doner weight in grams (*left panel*) and doner weight in grams per euro spent (*right panel*) as a function of time (visit number) across treatments NORMAL, COMPLIMENT and TIP

**Fig. 5:**
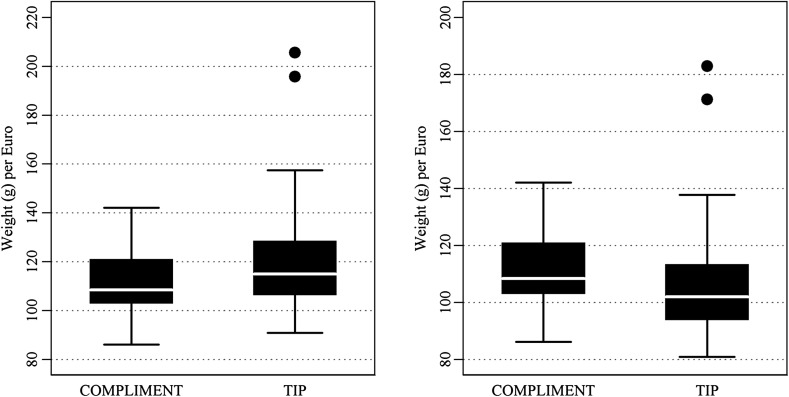
Normalized ice cream weight in grams (*left panel*) and in grams per euro (*right panel*) across treatments COMPLIMENT and TIP. Because of the normalization procedure mean weight of treatment NORMAL is set to 100

**Fig. 6:**
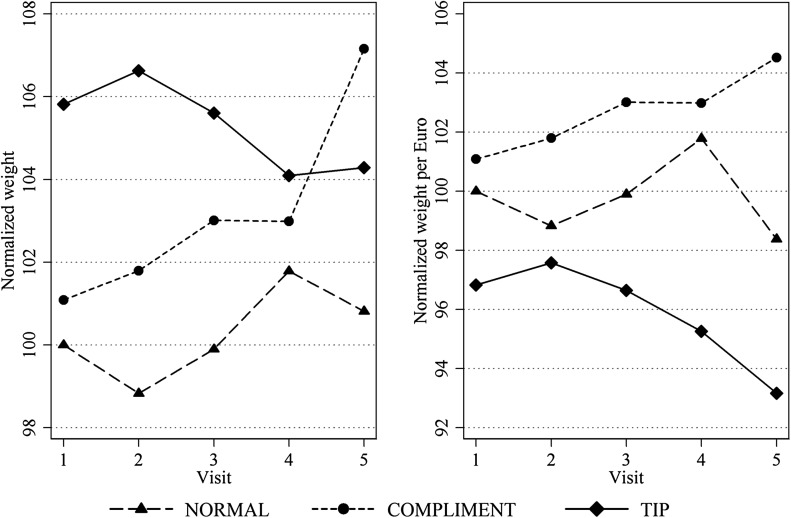
Normalized doner weight (*left panel*) and normalized doner weight per euro spent (*right panel*) as a function of time (visit number) across treatments NORMAL, COMPLIMENT and TIP

Table 4 presents descriptive statistics of raw doner weight in grams and doner weight in grams per euro spent, and Fig. 4 depicts treatment means for both measures (see Fig. 6 in the “Appendix” for a robustness check using first visit baseline-normalized weights). Across treatments, raw doner weight varies from 242 to 802 g, with a mean of 422 g. In NORMAL, raw weight remains relatively stable over time. In COMPLIMENT, doner weight is about 3 g higher in visit 1 and increases substantially over time, to a surplus over NORMAL of 23 g in visit 5. In TIP, raw weight is 17 g higher initially as well as in visit 5. Moreover, doner weight per euro is highest in treatment COMPLIMENT and is lower in treatment TIP than in treatment NORMAL.^14^^,^ 15

**Table 4:** Descriptive statistics: mean, median, standard deviation, minimum, and maximum of raw doner weights across treatments and over time in grams (top panel) and in grams per euro spent (bottom panel)

Treatment	Visit	Mean	Median	SD	Min	Max
*Raw doner weight* (g)						
NORMAL	1	413.35	409.05	60.14	295.15	681.55
	2	406.49	409.23	64.24	257.20	626.90
	3	409.88	398.60	58.64	314.45	646.25
	4	419.33	411.13	58.22	323.30	664.40
	5	416.15	410.73	71.89	244.90	698.45
COMPLIMENT	1	416.12	409.65	56.23	304.55	635.85
	2	418.94	416.30	60.69	241.65	649.15
	3	422.27	415.65	55.43	284.50	622.75
	4	422.35	419.45	57.59	276.90	589.10
	5	439.88	421.35	71.16	351.75	693.50
TIP	1	430.77	424.70	62.09	285.85	650.40
	2	435.67	427.10	61.24	293.70	633.65
	3	430.60	425.60	60.14	308.70	633.95
	4	427.68	414.38	65.13	325.55	643.50
	5	433.42	421.25	80.94	329.65	802.35
*Raw doner weight* (g) *per Euro spent*						
NORMAL	1	101.60	96.69	19.25	64.88	162.27
	2	100.81	95.83	18.14	58.81	149.26
	3	102.56	100.55	21.23	72.86	153.87
	4	105.25	99.29	20.23	64.66	158.19
	5	102.67	101.00	19.50	74.04	166.30
COMPLIMENT	1	102.32	99.69	18.62	65.36	151.53
	2	104.57	96.79	22.56	48.33	155.08
	3	105.59	99.19	21.11	73.42	151.22
	4	106.83	103.50	21.64	64.40	149.78
	5	108.66	108.92	19.82	75.02	163.48
TIP	1	96.46	94.09	15.17	66.10	141.39
	2	99.05	97.89	18.49	69.48	146.15
	3	98.27	95.99	17.15	68.28	137.82
	4	98.96	98.36	19.54	66.44	154.32
	5	98.69	99.84	20.94	60.42	174.42

Table 5 reports results from panel regressions with salesperson fixed effects (see Table 8 in the “Appendix” for a robustness check using first visit baseline-normalized weights). RAWWEIGHT serves as the dependent variable and we use binary treatment dummies for COMPLIMENT and TIP as independent variables (model 3). In model 4 we add time trends ($\hbox{TIME}\in \{1,2,3,4,5\}$) for each treatment to analyze whether the effects of immaterial and monetary gifts change over repeated visits. We also add experimenter and location dummy variables as well as variables AGE and FEMALE for salesperson age and gender. Standard errors are clustered at the salesperson level.

Focusing on raw doner weight, we find a significantly positive overall effect of both interventions compared to treatment NORMAL. The difference between treatments COMPLIMENT and TIP in model 3 is highly significant as well (Wald coefficient test, *F*(1,72) = 7.44, *p* = 0.0080). With regard to developments over time, we find that the time trend of treatment COMPLIMENT is significantly positive, with an average weight increase of 4.9 g per visit. The other treatments’ time trends are insignificant by themselves. Furthermore, while the difference in time trends between treatments NORMAL (with a positive and insignificant coefficient) and TIP (with a negative and insignificant coefficient) is not significant (Wald coefficient test, *F*(1,72) = 1.82, *p* = 0.1815), the trend difference between COMPLIMENT and TIP is highly significant (Wald coefficient test, *F*(1,72) = 7.46, *p* = 0.0079).

In contrast, our main result reverses when we investigate doner weight per euro spent. In model 3M in Table 5 we find qualitatively unchanged results for treatment COMPLIMENT, but marked differences for TIP. Here, we report a significantly negative overall effect of treatment TIP compared to NORMAL and COMPLIMENT. In particular, tipping yields 4.5 g less per euro spent compared to the baseline, while complimenting yields 2.6 g more per euro paid. Adding TIME in model 4M we again find a significantly positive time trend for treatment COMPLIMENT while no other coefficients remain significant. We provide further visit-level statistics on treatment effects in Table 9 in the “Appendix”.^16^

**Table 5:** Panel regressions of doner weight in grams (models 3, 4) and doner weight in grams per euro spent (models 3M, 4M) across treatments and over time

Regressors	Model 3	Model 4	Model 3M	Model 4M
TIP	19.183	27.350	−4.548	−2.740
	(3.963)***	(8.276)***	(0.953)***	(1.987)
COMPLIMENT	9.221	0.114	2.600	0.274
	(3.841)**	(6.762)	(0.997)**	(1.745)
NORMAL × TIME		1.644		0.406
		(1.580)		(0.400)
COMPLIMENT × TIME		4.942		1.248
		(1.489)***		(0.380)***
TIP × TIME		−1.274		−0.240
		(1.664)		(0.392)
Experimenter dummies		Yes		Yes
Constant	412.887	404.638	103.046	101.659
	(2.286)***	(5.882)***	(0.564)***	(1.417)***
*R* ^2^ within	0.05	0.07	0.10	0.12
*R* ^2^ between	0.03	0.01	0.06	0.33
*R* ^2^ overall	0.02	0.02	0.02	0.10
*N*	801	801	801	801

Panel regression with salesperson fixed effects. Standard errors clustered at the salesperson level (in parentheses)

* *p* < 0.1; ** *p* < 0.05; *** *p* < 0.01

Moreover, we can also analyze the data from a principal-agent perspective. The exchange of complimentary food for immaterial or monetary gifts may increase the utilities of the consumer and of the salesperson. Yet it is the principal (i.e., the owner of the restaurant) who pays the cost of the increased goods and material employed. At the same time, the principal may profit most from a satisfied customer, because the latter will be more likely to return and to spread the word among friends, thereby increasing the restaurant’s future sales. In the case where the owner herself serves as salesperson, she presumably derives the same direct utility as an employee salesperson from being tipped or complimented. Thus, the effect sizes in COMPLIMENT and TIP could differ for employee salespeople and owner salespeople, even though the direction of this difference is difficult to predict (Akerlof 1982).^17^

Fortunately, doner are frequently prepared not by employee salespersons, but by the restaurant owners themselves (the same is not true for in the ICECREAM setting). This allows us to study differential effects between employee and owner salespersons. As noted before, the final experimenter to interact with any particular salesperson inquired whether the salesperson was the owner of the restaurant. Since some salespersons changed during the experiment, we were able to obtain this information for all but 84 salespersons.

**Table 6:** Random effects panel regressions of doner weight in grams (models 3O, 4O) and doner weight in grams per euro spent (models 3MO, 4MO) across treatments and over time and including owner interactions

Regressors	Model 3O	Model 4O	Model 3MO	Model 4MO
TIP	22.033	36.754	−4.152	−1.109
	(6.195)***	(13.054)***	(1.531)***	(3.154)
COMPLIMENT	18.307	8.725	4.756	2.524
	(6.854)***	(9.998)	(1.715)***	(2.625)
NORMAL × TIME		1.161		0.379
		(2.275)		(0.587)
COMPLIMENT × TIME		4.675		1.274
		(2.050)**		(0.560)**
TIP × TIME		1.267		0.366
		(2.487)		(0.584)
Experimenter dummies		Yes		Yes
OWNER	−19.920	−24.942	−4.765	−3.980
	(14.518)	(17.501)	(5.037)	(4.436)
OWNER × TIP	−8.106	−23.120	−1.369	−4.409
	(8.354)	(17.674)	(2.039)	(4.291)
OWNER × COMPLIMENT	−14.519	−14.228	−3.337	−3.515
	(8.218)*	(14.202)	(2.141)	(3.744)
OWNER × NORMAL × TIME		0.585		−0.042
		(3.274)		(0.841)
OWNER × COMPLIMENT × TIME		0.093		−0.224
		(3.125)		(0.796)
OWNER × TIP × TIME		−4.553		−1.062
		(3.327)		(0.798)
Constant	420.664	408.506	104.720	93.579
	(9.889)***	(11.590)***	(3.343)***	(2.671)***
R2 within	0.05	0.07	0.12	0.14
R2 between	0.01	0.10	0.01	0.43
R2 overall	0.02	0.10	0.03	0.36
*N*	717	717	717	717

Standard errors clustered at the salesperson level (in parentheses)

* *p* < 0.1; ** *p* < 0.05; *** *p* < 0.01

In Table 6 we use a random effects specification and add interaction terms of OWNER and treatments as well as TIME, analyzing a potential owner effect for each treatment. We find that owners and employee salespersons react very similarly in all treatments. Although most coefficients are slightly negative, there is only one marginally significant result indicating that owners provide slightly less on average in the compliment treatment (see model 3O). However, this effect vanishes when including time effects for each treatment (model 4O). This implies that owners and salespersons are similarly affected by immaterial and monetary gifts.

The findings from both settings allow us to answer all research questions with results using the same numbering scheme as we used for the questions themselves.

*Result 1* Immaterial gifts in the form of compliments prior to the product’s preparation induce positive reciprocity by the salesperson.

*Result 2* A monetary gift, given prior to the product’s preparation, induces positive reciprocity as well. On the aggregate, the effect is more pronounced than following an immaterial gift before accounting for the cost of the gift. After accounting for the cost of the gift, results reverse, indicating that immaterial gifts are the strongest intervention when controlling for costs.

*Result 3* Immaterial gifts by the consumer lead to increasingly reciprocal behavior over time. Thus, compliments to the salesperson yield positive time effects compared to (1) reciprocity induced monetarily by tipping first, and compared to (2) normal orders.

## Conclusion

Reciprocation of monetary gifts is well-understood in economics. In contrast, there is little research on reciprocal behavior following immaterial gifts like compliments and how these effects develop over time. It is for this reason that we investigate how employees reciprocate after receiving immaterial or monetary gifts. Moreover, we investigate the stability of immaterial and monetary gifts over time with repeated gift exchange transactions.

We find that (1) immaterial gifts in the form of compliments significantly increase salespersons’ reciprocal behavior. Salespersons prepare ice cream and durum doner weighing more than ones obtained without intervention. We also report that (2) monetary gifts (tips) induce positive reciprocity which is on average stronger than that from immaterial gifts before costs. However, the increase in product weight does not necessarily suffice to compensate the customer for the increased cost of the tip, indicating that immaterial gifts are more effective when accounting for transaction costs. Finally, we show that (3) only reciprocation upon immaterial gifts grows significantly over repeated interactions, increasing by around 6 percent over the course of five visits. Reciprocal behavior conditional on monetary gifts, in contrast, does not vary significantly over time.

It is important to note that other factors than those discussed could have contributed to the reported effects. First, altruism among sellers could drive part of the results. The compliment and the tip could serve to update seller’s belief about how much the buyer values the product and, in case of an altruistic seller, could result in increased food weight. However, the effects of conditional altruism are difficult to quantify, particularly as conditional altruism and reciprocity are considered to be related concepts (Cox et al. 2008). Second, making a compliment may have the effect of reducing social distance, i.e., “the emotional proximity induced by the situation” (Charness and Gneezy 2008, p. 30). Such a reduction has been found to induce increased kindness (see for example Hoffman et al. 1996; Charness and Gneezy 2008). However, this effect would presumably also play a role in treatment NORMAL, thus limiting its impact on between-treatment comparisons. Third, part of the effects could be driven by guilt aversion (Charness and Dufwenberg 2006; Battigalli and Dufwenberg 2007; Ellingsen et al. 2010). Guilt aversion postulates that people feel guilty (and so incur a utility loss) whenever their behavior does not live up to their beliefs about the expectations of others. In our experiment, part of salespersons’ behavior could be motivated by guilt aversion in the face of customers’ kind acts of tipping and complimenting. As Ellingsen et al. (2010) point out, measuring guilt aversion is difficult even in a lab environment and so we cannot state the exact impact (if any) of guilt aversion. However, neither of the three explanations fully accounts for the effects found in treatment TIP and particularly in COMPLIMENT. They either cannot be measured or they would be expected to have a similar impact in all treatments.

To sum up, with our study we contribute to the existing literature along four dimensions. First, we explore reactions to immaterial and monetary gifts in the same two settings (ice cream and doner), making them comparable. We particularly consider the immaterial gift in the form of a compliment to constitute an important contribution to the literature. Compared to other studies, a private compliment like in our study is entirely immaterial, cannot be “stored”, cannot be used as physical evidence for impressing others, and thereby expresses respect directly (and usually only) to the recipient.

Second, we investigate both approaches in natural consumer–salesperson interactions in everyday life situations. We consider this an important aspect of our study. By studying immaterial and monetary gift exchange in customer–salesperson interactions, we test the robustness of the existing evidence from employer–employee interactions. We believe that customer–salesperson interactions are of high importance, because they occur very frequently (i.e., in some cases multiple times a day) and monetary and immaterial incentives (i.e., compliments) play a major role.

Third, we use a repeated setting to analyze how monetary and particularly immaterial gifts work over time. The increasingly reciprocal behavior upon repeated provision of immaterial gifts is a novel finding and adds to the literature which heretofore has focused only on a single workday, and on situations with a single (or double) exchange of monetary gifts (Gneezy and List 2006; Ockenfels et al. 2015). While the (initially higher) effects of material gifts do not exhibit a clear trend, the effects of immaterial gifts get stronger with repeated interactions. This observation suggests that immaterial forms of expressing approval may have stronger performance-reinforcing effects than money in the long run. An interesting avenue for future research would be to study the joint effect of immaterial and monetary gifts provided ex ante. We can only speculate, but expect that the joint effect is similar or even stronger than the effect of providing compliments alone. This, however, only holds as long as the monetary gift does not crowd out intrinsic motivations triggered by the compliment (see Bradler and Neckermann 2016 for some evidence on this question). We leave this issue open for future research.

Fourth, we investigate the robustness of our findings by analyzing whether our results replicate from the first to the second setting, i.e., from the ice cream to the doner setting.

Finally, we would like to emphasize that our results hold in a situation where the behavior exhibited in our treatments—while not entirely out of the ordinary—is not a general norm among consumers. We would expect our treatment effects to diminish with increasing frequency of tipping and complimenting, respectively, in the general consumer population. In the extreme case that tipping or complimenting were to become a social norm, we would expect negative reciprocity for consumers not providing an immaterial or monetary gift. In other words, we conjecture that consumers might be punished by the salesperson if no tip or compliment were given in advance when it is the norm to tip or compliment.

## Appendix

In an alternative approach to addressing salesperson-level fixed effects, we repeat our analyses using normalized ice cream and doner weights. To arrive at these normalized weights, we divide the weight of each ice cream cone or doner by the weight of the cone/doner bought from the same salesperson in (the first visit of) treatment NORMAL and multiply by 100, such that any deviation from 100 can be interpreted as a percentage difference relative to (the first visit in) treatment NORMAL.^18^

In the doner setting, the normalized weight can thus be calculated as:

1 $$\hbox{NORMWEIGHT}^\theta _{i,t} \,= \,\frac{W^\theta _{i,t}}{W^{\rm NORMAL}_{i,1}} \cdot 100.$$

Here, $W^\theta _{i,t}$ stands for doner weight in treatment $\theta \in \{\hbox{NORMAL},\,\hbox{COMPLIMENT},\,\hbox{TIP}\}$, purchased from salesperson *i* in visit *t*. To arrive at NORMWEIGHT we thus divide each doner’s weight $W^\theta _{i,t}$ by the weight of the first doner bought from the same salesperson in treatment NORMAL. This normalization eliminates salesperson and restaurant idiosyncratic effects and allows us to focus on treatment differences over time. For convenience, we multiply by 100, such that any deviation of NORMWEIGHT from 100 can be interpreted as a percentage difference relative to the NORMAL observation in the first visit. Equation 1 applies analogously to setting ICECREAM without the time indices *t*. In the case of normalized weight per euro, we divided the raw weights by the price paid before applying Eq. 1.

### Setting ice cream

Figure 5 displays NORMWEIGHT in the same format as does Fig. 2 in the body of the paper. Since weights are normalized to the weight observed for the same salesperson in treatment NORMAL, we do not print a box for normalized weight in this treatment as it equals 100. The figure shows a similar picture as does Fig. 2, underlining the robustness of our choice of analysis approach.

We next turn to OLS regressions to study NORMWEIGHT. Table 7 lists results which correspond to those of Table 2 in the body of the paper. Since we normalize weights, we can forego the use of salesperson fixed effects and can include salesperson-specific characteristics like gender (FEMALE) and median experimenter-estimated age (AGE) in the regression. The results confirm the robustness of our findings.

**Table 7:** OLS regressions of normalized ice cream cone weight (models 1N, 2N) and of normalized weight per euro spent (models 1MN, 2MN) across treatments

Regressors	Model 1N	Model 2N	Model 1MN	Model 2MN
COMPLIMENT	11.318	11.318	11.318	11.318
	(2.241)***	(2.303)***	(2.241)***	(2.286)***
TIP	21.891	21.891	7.773	7.773
	(4.163)***	(4.211)***	(3.657)**	(3.714)**
Experimenter dummies		yes		yes
FEMALE		−0.651		−0.452
		(3.897)		(3.563)
AGE		−0.098		−0.092
		(0.227)		(0.206)
Constant	100.000	103.359	100.000	102.822
	(0.000)***	(8.361)***		(7.573)***
*R* ^2^	0.24	0.24	0.10	0.10
Adj. *R* ^2^	0.22	0.20	0.08	0.05
*N*	108	108	108	108

Standard errors clustered at the salesperson level (in parentheses)

* *p* < 0.1; ** *p* < 0.05; *** *p* < 0.01

### Setting DONER

Figure 6 displays NORMWEIGHT in the DONER setting in the same format as does Fig. 4 in the body of the paper. It documents that our main findings are robust to the use of normalized weights and graphically underlines the finding of a weakly significant, negative trend in doner weight per euro in treatment TIP.

Table 8 lists results for NORMWEIGHT which correspond to those of Table 5 in the body of the paper. Again, since we normalize weights, we can forego the use of salesperson fixed effects and can include salesperson-specific characteristics like gender (FEMALE) and median experimenter-estimated age (AGE) in the regression. The results confirm the robustness of our findings.

**Table 8:** OLS regressions of normalized doner weight in grams (models 3N, 4N) and in grams per euro spent (models 3MN, 4MN) across treatments and over time

Regressors	Model 3N	Model 4N	Model 3MN	Model 4MN
COMPLIMENT	2.724	0.509	2.698	0.571
	(1.037)**	(1.699)	(1.034)**	(1.701)
TIP	5.253	7.901	−3.665	−1.240
	(1.041)***	(2.090)***	(0.934)***	(1.925)
NORMAL × TIME		0.481		0.064
		(0.446)		(0.444)
COMPLIMENT × TIME		1.285		0.837
		(0.393)***		(0.402)**
TIP × TIME		−0.465		−0.802
		(0.449)		(0.419)*
Experimenter dummies		Yes		Yes
FEMALE		−0.211		−0.237
		(3.241)		(3.144)
AGE		−0.065		−0.054
		(0.132)		(0.127)
Constant	100.173	101.606	99.794	102.065
	(0.975)***	(5.318)***	(0.967)***	(5.118)***
*R* ^2^	0.03	0.06	0.04	0.08
Adj. *R* ^2^	0.03	0.04	0.04	0.06
*N*	797	797	797	797

Standard errors clustered at the salesperson level (in parentheses)

* *p* < 0.1; ** *p* < 0.05; *** *p* < 0.01

The differences between treatments over time are summarized in more detail in Table 9. It reports the results of paired *t* tests for treatment differences of raw and normalized doner weights and of raw and normalized doner weights per euro spent, for each visit.^19^ We find a general, positive effect of treatment TIP before accounting for costs. Mean product weight is significantly higher in treatment TIP than in treatment NORMAL in 4 out of 5 visits. Comparing the effects of monetary and immaterial gifts is less clear. During the first two visits the difference is significantly positive, but it declines monotonically and reverses (although insignificantly) for the last visit. Regarding the effects on weight per euro, we find that the comparison between treatments NORMAL and COMPLIMENT is essentially unaffected, while TIP yields significantly lower weight per euro than either NORMAL and COMPLIMENT in every visit.

**Table 9:** Pairwise treatment differences in raw doner weight and in normalized doner weight (denoted by “N” in the first column and shown in the lower panel) and in raw and normalized weight per euro

Visit	Raw weight in g			Raw weight in g per euro		
	COMPLIMENT versus NORMAL	TIP versus NORMAL	TIP versus COMPLIMENT	COMPLIMENT versus NORMAL	TIP versus NORMAL	TIP versus COMPLIMENT
1	0.49	20.10	18.30	0.10	−4.65	−5.07
	(6.48)	$$(6.99)^{***}$$	$$(5.42)^{***}$$	(1.70)	$$(1.68)^{***}$$	$$(1.41)^{***}$$
2	13.30	29.40	15.00	4.31	−1.51	−6.10
	(8.49)	$$(7.59)^{***}$$	$$(7.56)^{*}$$	$$(2.22)^{*}$$	(1.85)	$$(1.77)^{***}$$
3	12.10	16.60	7.31	3.02	−5.49	−7.89
	$$(5.92)^{**}$$	$$(6.11)^{***}$$	(6.81)	$$(1.46)^{**}$$	$$(1.51)^{***}$$	$$(1.67)^{***}$$
4	−1.01	6.03	5.46	0.05	−7.76	−8.12
	(6.26)	(7.11)	(6.76)	(1.65)	$$(1.73)^{***}$$	$$(1.73)^{***}$$
5	22.50	17.40	−7.93	5.76	−4.37	−10.80
	$$(8.47)^{**}$$	$$(7.07)^{**}$$	(8.76)	$$(2.09)^{***}$$	$$(1.77)^{**}$$	$$(2.03)^{***}$$
1N	1.09	5.82	4.33	1.09	−3.17	−4.61
	(1.61)	$$(1.74)^{***}$$	$$(1.44)^{***}$$	(1.61)	$$(1.59)^{*}$$	$$(1.39)^{***}$$
2N	3.57	7.58	3.74	3.57	−1.46	−5.28
	$$(2.10)^{*}$$	$$(1.87)^{***}$$	$$(1.87)^{**}$$	$$(2.10)^{*}$$	(1.75)	$$(1.79)^{***}$$
3N	2.89	3.98	1.76	2.89	−4.93	−7.14
	$$(1.49)^{*}$$	$$(1.41)^{***}$$	(1.62)	$$(1.49)^{*}$$	$$(1.36)^{***}$$	$$(1.55)^{***}$$
4N	0.17	1.83	1.29	0.17	−7.00	−7.51
	(1.53)	(1.82)	(1.70)	(1.53)	$$(1.65)^{***}$$	$$(1.61)^{***}$$
5N	5.82	4.00	−2.52	5.68	−4.56	−10.92
	$$(2.15)^{***}$$	$$(1.70)^{**}$$	(2.02)	$$(2.14)^{**}$$	$$(1.62)^{***}$$	$$(1.96)^{***}$$

Paired *t* tests are run to test for differences between treatments

Standard errors in parentheses. For instance, the value of 0.49 in the first cell of the treatment comparison COMPLIMENT versus NORMAL (first column) is calculated as doner weight in COMPLIMENT minus doner weight in NORMAL, indicating a weight 0.49 g higher in COMPLIMENT than in NORMAL in visit 1

*, ** and *** represent the 10, 5 and 1% significance levels of two-sided, paired *t* tests

## Footnotes

1 In related research Masclet et al. (2003) show that immaterial expressions of *disapproval* can serve as a form of punishment, which leads punished parties to increase their future contributions to a public good.

2 In a study more loosely related to ours, Grant and Gino (2010) report positive effects of expressions of gratitude on prosocial behavior in a field study on university fund raising. In their Experiment 3 the director visited the organization to thank the fundraisers for their work. The authors show that the number of voluntary calls by the fundraisers exposed to this gratitude treatment increased after the intervention, indicating prosocial behavior. Importantly, although the intervention is of immaterial type, the authors do not focus on reciprocal, but on prosocial behavior.

3 A doner durum is a dish of Turkish origin, made of meat roasted on a vertical spit, and served in a wrap. The dish is referred to as “shawarma” in Arabic, as “doner” in German and Turkish, as “gyros” in Greek, and as “gyro” in the US. Doner durum are a popular form of snack particularly, but not exclusively, in Europe. The annual revenues of the doner industry in Germany (the United Kingdom) amounted to € 3.5bn in 2011 (£ 2.2bn, as of December 2015). See http://online.wsj.com/news/articles/SB10001424052702304432704577350194262835880 and http://www.britishkebabawards.co.uk/about_british_kebab_awards, retrieved on December 4, 2015.

4 The model of Levine (1998) offers similar predictions and can serve as motivation as well. The underlying idea is that altruistic individuals reciprocate in a more altruistic manner when they believe they interact with other altruists.

5 If, for example, the price was €0.90 and the experimenter tipped €0.10, he would not hand over a single 1 Euro coin but would instead hand over one 50 Cent coin, two 20 Cent coins and one 10 Cent coin.

6 This procedure was inspired by the randomization in the credence goods field experiment of Balafoutas et al. (2013).

7 As one reviewer points out, due to our experimental design we are essentially even using salesperson-place-time fixed effects.

8 See Table 7 in the “Appendix” for a robustness check using baseline-normalized weight.

9 Note that observing reciprocity over time in the ice cream setting is impossible because of unsystematic working hours, job rotation, and a large share of part-time workforce in the branches of the fast food chain.

10 9 observations were lost due to technical problems.

11 In 14 of the 54 restaurants the salesperson changed at least once during the elicitation period. We still visited each restaurant 15 times, but recorded the observations as stemming from different salespersons and controlled for this in our analysis.

12 Experimenter arrivals at the different shops were not randomized systematically, since the experimenters operated independently after the first visit (during the first visit, they entered each restaurant separated by short time intervals, such that they could discuss the salesperson’s appearance to make sure each experimenter interacted with the same salesperson throughout).

13 Conditional on the experimenter’s caloric requirements, in some cases the product was also consumed directly by the experimenter himself (after the weighing procedure).

14 A critique of the weight variable specifically in the DONER setting could be that ingredients have different economic values which are not perfectly linearly related to their prices. However, it is unrealistic to assume that a salesperson wishing to reciprocate a perceived kindness would only add one or two cheaper ingredients, thereby deviating from the ‘optimal’ input composition, making the product less tasty. Furthermore, the one ingredient whose weight is fixed, the bread wrapper, is relatively cheap, especially compared to the main ingredient, meat. This implies that any extra food amount provided is made up of relatively more expensive ingredients.

15 Our results are affected by a simultaneous price raise of €0.50 by 7 shops in visit 5. Assuming prices would have remained stable, visit 5 mean weights per euro would have been 105.72 in NORMAL, 111.95 in COMPLIMENT and 101.44 in TIP.

16 It is also important to mention that our analyses measure only a lower bound of the extra service provided in treatments TIP and particularly COMPLIMENT. The experimenters frequently received additional benefits which could not enter into our analysis. In treatment COMPLIMENT the experimenters received 16 rebate cards, free servings of tea, soft drinks, almond juice and prawn crackers, in one instance two rebate marks instead of one, and a return compliment. In treatment TIP the experimenters received a total of 9 rebate cards, a serving of special meat for a tasting, and repeated free servings of tea and prawn crackers. In treatment NORMAL the experimenters received only 12 rebate cards and, once, two rebate marks.

17 Note that the issue is in fact more complex than suggested here. Assume that only the employee serves customers in a given restaurant. Even in this case, both the owner and the employee may profit from reciprocation. Leaving a positive impression with the customer by reciprocating his kind gesture (tip or compliment) may increase the likelihood of the customer returning to the restaurant or spreading the word among her friends and relations. This in turn increases the likelihood for the salesperson to obtain future tips or compliments from the customer or her friends, and for the owner of reaping the benefits of increased future revenue. Thus if the salesperson in this situation decides to reciprocate and the owner considers the expected impact on future revenues to outweigh her cost for the increased goods and materials employed, there is no agency conflict. Similarly, if the owner considers the extra costs to outweigh the benefits from reciprocating, and the salesperson decides to refrain from reciprocating for fear of hurting the restaurant owner, there is no agency conflict. Agency conflicts arise whenever the salesperson chooses to reciprocate even though the owner would not want her to, or if the salesperson does not reciprocate if the owner would want her to. It is even possible that the owner would not want the employee salesperson to reciprocate while the owner herself, in the same situation, reciprocates. This is because, when the owner is serving the customer herself, she herself experiences the “warm glow” (Andreoni 1989) from having been kind to the customer, which may outweigh extra costs which are not compensated by sufficient positive future benefits.
